# Structural and Functional Progression in Open-Angle Glaucoma with Unilateral Peripapillary Intrachoroidal Cavitation

**DOI:** 10.3390/jcm15062139

**Published:** 2026-03-11

**Authors:** Kaho Akiyama, Shuichiro Aoki, Shiroaki Shirato, Rei Sakata, Makoto Aihara, Megumi Honjo, Hitomi Saito

**Affiliations:** 1 Department of Ophthalmology, University of Tokyo Graduate School of Medicine, 7-3-1 Hongo, Tokyo 113-8655, Japan; kahopatchouli6028@gmail.com (K.A.);; 2 Yotsuya Shirato Eye Clinic, 2-6 Samoncho, Tokyo 160-0017, Japan

**Keywords:** high myopia, glaucoma, optic neuropathy, visual field, circumpapillary retinal nerve fiber layer thickness, peripapillary atrophy, longitudinal study

## Abstract

**Background/Objectives**: The aim of this study was to investigate the longitudinal visual field (VF) and circumpapillary retinal nerve fiber layer thickness (cpRNFLT) changes in open-angle glaucomatous (OAG) participants with unilateral peripapillary intrachoroidal cavitation (PICC) and to identify factors associated with VF progression. **Methods**: Sixty eyes of 30 OAG patients with unilateral PICC were included in this retrospective longitudinal observational study. Humphrey 24–2 VF testing and optical coherence tomography scanning were performed in all eyes over a period exceeding 5 years. VF progression was assessed using mean deviation (MD) and superior and inferior total deviation (TD) slopes. Structural progression was evaluated using global, superior, and inferior cpRNFLT thinning rates. Longitudinal changes were compared between PICC eyes and their contralateral non-PICC eyes. Factors associated with superior or inferior TD slopes were analyzed using linear mixed-effects models. The following variables were included as explanatory variables: age, sex, intraocular pressure, axial length, Bruch’s membrane opening (BMO) and scleral flange opening (SFO) area, SFO/BMO offset magnitude, disk tilt, disk rotation, baseline superior or inferior TD, baseline corresponding cpRNFLT, and the presence of PICC. **Results**: MD slope was −0.24 ± 0.35 dB/year in PICC eyes and −0.35 ± 0.53 dB/year in contralateral eyes. There was no significant difference in MD slope, superior and inferior TD slope, or the rate of cpRNFLT thinning (all *p* > 0.05). In multivariable analysis, the presence of PICC was associated with slower progression in the corresponding superior VF (*p* = 0.037), whereas greater SFO/BMO offset magnitude was associated with faster progression (*p* = 0.047). **Conclusions**: OAG eyes with PICC exhibited modest functional and structural progression over 5 years, comparable to that of contralateral non-PICC eyes. The presence of PICC was associated with slower corresponding superior VF progression, whereas greater myopia-associated structural change was related to faster progression. Our findings characterize the clinical course of eyes with pronounced myopic ONH deformation, highlighting the importance of detailed ONH structural assessment in the management of myopic glaucoma.

## 1. Introduction

Myopia is a well-established risk factor for open-angle glaucoma (OAG), as consistently shown in large epidemiologic studies [1,2]. However, whether myopia is also associated with glaucoma progression remains controversial [2]. Some highly myopic (HM) eyes reportedly show no structural or visual field (VF) progression over time [3], and several reports have suggested slower progression in OAG eyes with HM compared with those without HM [4,5]. These observations have been linked to distinct optic nerve head (ONH) structural changes induced by axial length (AL) elongation [6,7], which may alter the mechanisms and clinical characteristics of VF loss beyond typical intraocular pressure (IOP)-related glaucomatous optic neuropathy (GON) [8]. Since structural and functional progression in OAG eyes with distinct myopic structural changes may differ from that in typical GON, evaluating their longitudinal changes is clinically important.

Peripapillary intrachoroidal cavitation (PICC) is one such HM-associated ONH structural abnormality characterized by a hyporeflective space within the choroid observed on optical coherence tomography (OCT) and a yellow-to-orange lesion adjacent to the ONH [9,10,11]. Previous reports have indicated that 20.8–73.3% of PICC eyes are accompanied by VFDs [6,12,13,14,15,16,17,18]. Our recent study reported that myopia-induced tissue disruptions [11] were associated with location-corresponding sensitivity reduction in PICC eyes in addition to circumpapillary retinal nerve fiber layer thickness (cpRNFLT) thinning typically observed in glaucoma [19]. These findings suggest that VFDs in PICC eyes may involve mechanisms distinct from those of conventional glaucoma. However, their long-term structural and functional course has not yet been clarified.

Our recent work also showed that PICC eyes exhibit pronounced ONH structural alterations, including enlargement of the Bruch’s membrane opening (BMO), expansion of the peripapillary (PPA)-gamma and delta regions, and greater optic disk tilt [20]. The severity of these myopic structural changes has been linked to the progression of visual field (VF) loss in HM and glaucoma [21,22,23,24]. Evaluating whether and how these ONH characteristics influence VF progression in PICC eyes is therefore of significant clinical relevance.

We hypothesized that structural and functional progression in PICC eyes diagnosed as OAG would differ from that of typical glaucoma and that ONH structural changes associated with PICC formation would contribute to VF progression. Since PICC does not develop in all highly myopic eyes and can present unilaterally even under comparable genetic and environmental conditions, unilateral PICC provides a unique internal control model that minimizes interindividual variability. To further minimize potential confounding, we excluded eyes with pathological myopia, which often presents with retinal lesions that can directly affect VF test results. In this study, we aimed to investigate the longitudinal structural and functional changes in OAG eyes with PICC by comparing PICC eyes and their contralateral eyes in unilateral PICC participants without pathological myopia and to identify factors associated with VF progression.

## 2. Materials and Methods

### 2.1. Participants

Protocols for this observational study were approved by the Japan Medical Association (approval ID: R6–19). The need for informed consent was waived due to the retrospective nature of the study.

Consecutive OAG patients who visited Yotsuya Shirato Eye Clinic (Tokyo, Japan) for regular eye examinations between January and March 2020 were retrospectively reviewed. Among these consecutive cases, patients with unilateral PICC were enrolled. Clinical records available through March 2025 were reviewed for analysis. Inclusion criteria for this study were (1) diagnosis of OAG in both eyes, (2) presence of unilateral PICC, and (3) at least 5 follow-up VF and OCT tests obtained over >5 years at 3–6-month intervals. Exclusion criteria for this study were (1) prior intraocular surgery other than uncomplicated cataract surgery; (2) clinically significant cataract or corneal opacity leading to impaired image quality or segmentation errors on OCT; and (3) non-glaucomatous optic neuropathy, retinal disease, or pathological myopia, including diffuse or patchy chorioretinal atrophy, macular atrophy, myopic choroidal neovascularization, lacquer cracks, Fuchs’ spot, or posterior staphyloma [25] in either eye. Unreliable VF measurements (≥33% fixation loss, ≥33% false negative errors, or ≥33% false positive errors) and poor OCT scans with insufficient image quality, defined as a signal strength index < 8, as well as those containing artifacts or segmentation errors [26], were excluded from the analysis.

### 2.2. Baseline Ocular Examinations

A comprehensive baseline ophthalmic evaluation was performed in all participants, including refraction and corneal curvature radius measurements, measurement of best corrected visual acuity, AL measurements (IOLMaster, Carl Zeiss Meditec, Dublin, CA, USA), intraocular pressure (IOP) measurements with Goldmann applanation tonometry, slit-lamp examination, gonioscopy, fundus examination under pupillary dilation including stereoscopic assessment of ONH, OCT imaging (Cirrus HD-6000, Carl Zeiss Meditec, Dublin, CA, USA), fundus photography (TRC-NW8F, Topcon, Tokyo, Japan), and VF testing using the Humphrey field analyzer (Carl Zeiss Meditec, Dublin, CA, USA) with the 24-2 Swedish Interactive Threshold Algorithm standard protocol.

OAG was diagnosed in eyes showing open angles on gonioscopy and characteristic glaucomatous ONH changes, including neuroretinal rim narrowing or notching and retinal nerve fiber layer defects, based on fundus examination and stereophotographic assessment, with these structural glaucomatous changes also confirmed as corresponding retinal nerve fiber layer defects on OCT. VF results were considered abnormal when at least one of the following Anderson–Patella criteria [27] was met: (1) the pattern deviation probability plot showed a cluster of 3 or more points with a probability of less than 5% and at least 1 point with a probability of less than 1% in an expected location; (2) the pattern standard deviation had a probability of less than 5%; or (3) the glaucoma hemifield test indicated that the field was out of normal limits. Each eye was diagnosed independently by two glaucoma specialists (H.S. and K.A.), and discrepant cases were adjudicated by a third reviewer (S.A.).

**Figure 1:**
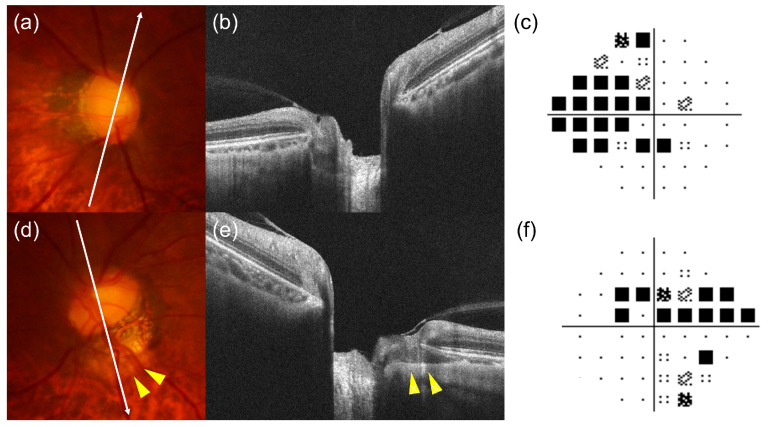
(**a**,**d**) Fundus photographs, (**b**,**e**) optical coherence tomography (OCT) B-scan images obtained from optic nerve head-centered radial scans, and (**c**,**f**) the pattern deviation plots of the 24-2 Humphrey visual field test of a participant with unilateral peripapillary intrachoroidal cavitation (PICC). The white arrows in panels (**a**,**d**) indicate the location and orientation of the corresponding OCT B-scans shown in panels (**b**,**e**). PICC was identified only in the left eye and appeared as a yellow lesion at the margin of peripapillary atrophy ((**d**), yellow arrowhead) and as an intrachoroidal hyporeflective space on OCT B-scan ((**e**), yellow arrowhead).

PICC was identified on OCT images by the presence of hyporeflective regions accompanied by posterior bowing of the peripapillary sclera, using twelve ONH-centered 6.0 mm radial scans and 200 × 200 ONH cube scans acquired with the Cirrus HD-6000 OCT system (Carl Zeiss Meditec, Dublin, CA, USA) (Figure 1) [9,11,12]. OCT images were independently evaluated for the presence of PICC by two experienced examiners (K.A., H.S.), and disagreements were resolved by a third adjudicator (S.A.). PICC was considered present when the lesion was identified in two or more consecutive ONH-centered radial OCT scans. The PICC location was classified as superior or inferior according to the radial scans.

### 2.3. Optic Nerve Head Structure Parameters

**Figure 2:**
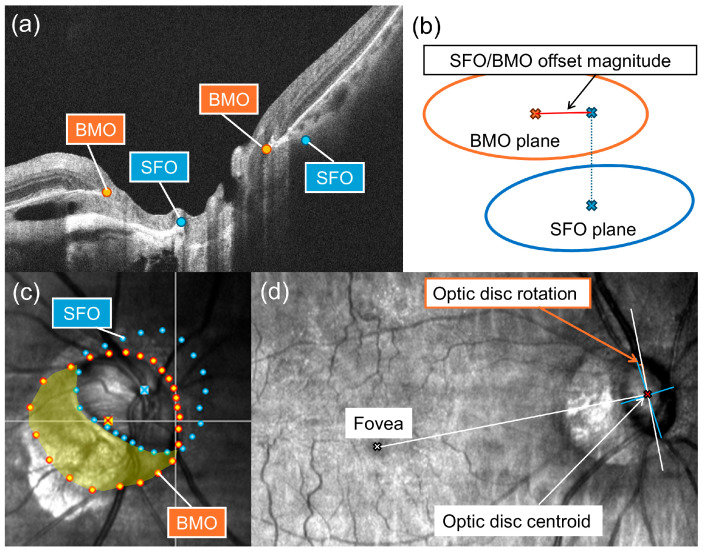
Identification of deep optic nerve head (ONH) structures. (**a**) Bruch’s membrane opening (BMO; orange dots) and scleral flange opening (SFO; blue dots) were identified on twelve radial optical coherence tomography B-scans. (**b**) SFO/BMO offset magnitude was defined as the distance between the centroids of BMO and SFO after projection onto the BMO plane (red line). (**c**) Superimposition of BMO and SFO points on a line scanning laser ophthalmoscopy (LSLO) image. The temporal region between BMO and SFO corresponds to a peripapillary zone without Bruch’s membrane and an area of exposed scleral flange (yellow area). (**d**) Assessment of optic disk morphology. The optic disk centroid (red cross), along with the longest and shortest optic disk diameters (blue lines) were defined based on the clinical disk margin on the LSLO image. Optic disk rotation was defined as the angle between the longest axis and the vertical meridian, which was set perpendicular to the line connecting the optic disk centroid and fovea (white cross).

A total of twelve ONH-centered radial B-scans, separated by 15°, were exported and analyzed using Fiji ImageJ 1.54f (Java 1.8.0_322 64-bit, National Institutes of Health, Bethesda, MD, USA). BMO and scleral flange opening (SFO) points were manually identified on both sides of each radial B-scan by two experienced examiners (K.A., H.S.) (Figure 2).

SFO is a recently proposed anatomical landmark corresponding to the neural canal opening. On OCT images, SFO was identified as the transition point between the anterior laminar surface and the anterior scleral flange surface when the scleral flange was present [28,29]. In areas where the scleral flange was not exposed, the SFO location was considered equivalent to the anterior scleral canal opening (ASCO) location, in accordance with previous reports [29]. Coordinates of BMO and SFO were exported and analyzed using R software version 4.5.1 (R Foundation for Statistical Computing, Vienna, Austria). For each structure, a reference plane minimizing the mean squared axial error was fitted to the corresponding 24 landmark points. The coordinates were subsequently projected onto these planes, and best-fit ellipses were generated to determine the centroids, areas, and longest and shortest diameters. Ovality of the BMO and SFO was calculated as the ratio of the longest to the shortest diameter derived from each ellipse. The SFO/BMO offset magnitude was used as an index of relative displacement between the two structures and was calculated as the distance separating the centroids of the BMO and SFO after projection onto the BMO plane (Figure 2b) [30]. ASCO is defined as the intersection of the anterior scleral surface and the border tissue [28], but it cannot be reliably identified in PICC regions with disrupted border tissue. Therefore, SFO was used to represent the neural canal opening instead of ASCO. The temporal region located between the BMO and SFO encompasses the combined area of PPA-gamma and PPA-delta. PPA-gamma is characterized by the absence of Bruch’s membrane, whereas PPA-delta represents a region with an elongated and thinned peripapillary scleral flange lacking choroidal tissue [31]. Enlargement of the SFO/BMO offset magnitude reflects expansion of this combined PPA-gamma and PPA-delta region (Figure 2c) [29]. Excellent reproducibility of BMO- and SFO-related measurements, both within each of the observers (K.A.) and between observers (K.A. and H.S.), has been reported previously, with coefficients ranging from 0.966 to 0.994 [19].

Clinical disk margins were manually delineated on line scanning laser ophthalmoscopy images derived from 200 × 200 macular cube OCT scans with Fiji ImageJ. A best-fit ellipse was applied, and the optic disk centroid, area, and the longest and shortest diameters were determined. Disk tilt was quantified using the ovality index, calculated as the ratio of the longest to the shortest disk diameter. Disk rotation was evaluated by measuring the angle formed between the longest disk axis and a vertical reference line. This vertical reference was set perpendicular to the line connecting the optic disk centroid and the fovea (Figure 2d). Inferotemporal orientation was assigned to positive values of disk rotation, whereas supranasal orientation corresponded to negative values.

### 2.4. Circumpapillary Retinal Nerve Fiber Layer Thickness and Choroidal Thickness

Circumpapillary retinal nerve fiber layer thickness (cpRNFLT) was measured on a 3.46 mm BMO-centered circle, and cpChT on a 4.5 mm circle. Global, superior, and inferior cpRNFLT values were obtained from ONH cube scan data following automatic segmentation with custom Zeiss software (version 11.5), while cpChT measurements were performed manually on individual radial scans using Fiji ImageJ.

### 2.5. Magnification Correction

Correction for ocular magnification was applied to all planar measurements, including BMO and SFO area, optic disk area and diameters, as well as to the measurement circle diameters for cpRNFLT and cpChT [32,33].

### 2.6. Statistical Analysis

All data are expressed as mean ± standard deviation unless otherwise indicated. Baseline background parameters, ONH parameters, VF, and cpRNFLT were compared between PICC eyes and their contralateral eyes. Normality of paired differences was assessed with the Shapiro–Wilk test, after which paired *t*-tests or Wilcoxon signed-rank tests were applied to normally and non-normally distributed variables, respectively. The McNemar test was used to compare categorical variables.

Progression between PICC eyes and their contralateral eyes was compared by calculating the mean deviation (MD) slope, superior and inferior total deviation (TD) slope, and the rate of change for global, superior, and inferior cpRNFLT. The superior and inferior TD were determined by averaging the TD measurements from the superior and inferior hemifields, respectively. Progression of MD as well as superior and inferior TD in each eye were identified when linear regression demonstrated a significantly negative slope (*p* < 0.05) for an individual eye. Since the PICC location was classified as superior or inferior, factors associated with VF progression were evaluated separately for superior and inferior TD slopes using univariable and multivariable linear mixed-effects models with a random intercept for each participant to account for the correlation between fellow eyes. Age, sex, IOP at the time of OCT imaging, AL, BMO area, SFO area, SFO/BMO offset magnitude, disk tilt, disk rotation, baseline superior or inferior TD, baseline corresponding cpRNFLT, and the presence of PICC were selected as explanatory variables. Absence of significant multicollinearity among the explanatory variables was confirmed by examining variance inflation factors. All possible combinations of linear mixed-effects models were constructed, and the best-fit model based on the second-order bias-corrected Akaike Information Criterion index was selected for discussion. Statistical analyses were performed with R software version 4.5.1.

## 3. Results

Among consecutive OAG patients examined between January and March 2020, a total of 100 eyes of 50 OAG participants with unilateral PICC initially met the inclusion criteria. After the exclusion of 20 participants (13 with prior glaucoma surgery, 5 with retinal disease, and 2 with pathological myopia or suspected pathological myopia), 60 eyes of 30 participants were included in the current study. Table 1 presents baseline characteristics of the participants and the intra-individual comparison of ONH structure parameters, baseline VF, and cpRNFLT between PICC eyes and their contralateral eyes. PICC eyes showed significantly greater AL, BMO area, SFO/BMO offset magnitude, and optic disk tilt than their contralateral eyes (*p* = 0.010, *p* < 0.001, *p* < 0.001, *p* < 0.001, respectively). There were no significant differences in visual acuity, IOP, disk rotation, baseline VF, cpRNFLT, and cpChT between the two groups. All PICC eyes exhibited PICC in the inferior region.

Table 2 presents the functional and structural progression and its comparison between PICC eyes and their contralateral eyes. MD slope was −0.24 ± 0.35 dB/year in PICC eyes and −0.35 ± 0.53 dB/year in contralateral eyes. No significant differences were observed in MD slope, superior or inferior TD slope, or the rate of global, superior, or inferior cpRNFLT thinning (all *p* > 0.05). The number of eyes with progression also did not differ significantly between the two groups. However, the prevalence of superior TD progression, which corresponds to inferiorly located PICCs, was approximately half in PICC eyes compared with their contralateral eyes.

Table 3 presents the effects of baseline characteristics, ONH parameters, VF parameters, corresponding cpRNFLT, and the presence of PICC on the superior and inferior TD slopes. In the multivariable model, the presence of PICC was significantly associated with slower superior TD progression (*p* = 0.037), whereas a greater SFO/BMO offset magnitude was associated with faster superior TD progression (*p* = 0.047). Female sex (*p* = 0.042) and thinner baseline superior cpRNFLT (*p* < 0.001) were significantly associated with faster inferior TD progression.

## 4. Discussion

In this 5-year follow-up study of OAG participants with unilateral PICC, 13.3% of PICC eyes showed significant global VF progression, and the rates of VF and cpRNFLT progression were comparable to those in their contralateral non-PICC eyes. In multivariable analysis, the presence of PICC was associated with slower progression in the superior VF, whereas greater ONH misalignment was associated with faster progression. To the best of our knowledge, this is the first study to characterize the longitudinal course of OAG eyes with PICC.

**Figure 3:**
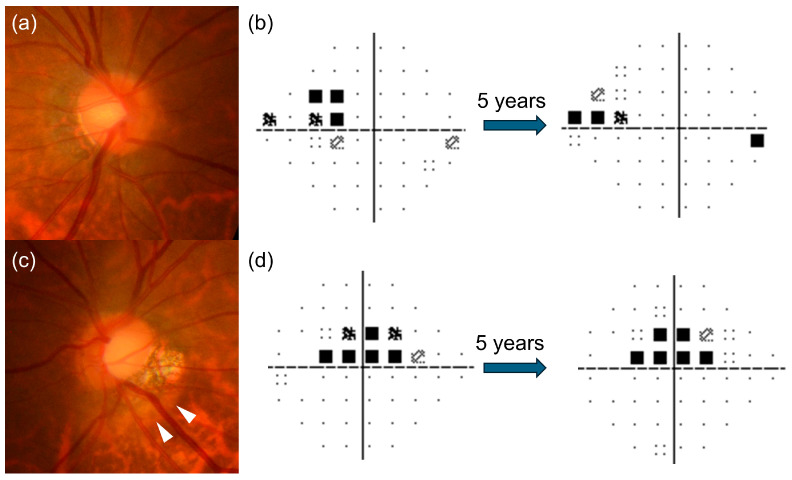
(**a**,**c**) Fundus photographs from a participant with unilateral peripapillary intrachoroidal cavitation (PICC, white arrowheads) and (**b**,**d**) the pattern deviation plots of the 24-2 Humphrey visual field test at the baseline (**left**) and after 5-year follow-up (**right**). The visual field defects remained stable in both eyes during the 5-year follow-up period.

In our present cohort, the mean MD slope in PICC eyes was −0.24 ± 0.35 dB/year, which appears relatively mild compared with previously reported rates in large cohort studies of treated OAG, ranging from −0.62 to −0.05 dB/y [34,35,36,37]. The rate of global cpRNFLT thinning was −0.15 ± 1.17 µm/y, which is considerably slower than the reported structural progression in HM OAG eyes, ranging from −1.17 to −0.54 µm/y [38,39], and within the range expected for age-related thinning in healthy HM eyes of approximately −0.44 µm/y [39]. Some previous studies have suggested that HM OAG eyes may progress more slowly than non-myopic or mild-to-moderate myopic OAG eyes [3,4,5]. Doshi et al. reported no VF progression in eyes with myopic changes over a follow-up period of up to 7 years, regardless of the use of IOP-lowering treatment [3]. Nitta et al. showed that HM OAG eyes had a significantly slower MD slope (−0.19 ± 0.28 dB/year) than non-myopic OAG eyes (−0.38 ± 0.55 dB/year), indicating that glaucomatous progression in HM eyes may follow a distinct course from that observed in typical OAG [5]. Regarding the progression of OAG eyes with PICC, previous evidence has been limited mainly to case reports and small case series. Both stable cases and cases showing progression have been reported [40,41], but the long-term clinical course has not been well characterized. Approximately 10% of our OAG eyes with PICC demonstrated significant progression, but both functional and structural changes were modest over 5 years, which may partly account for the slow progression reported in a subset of HM OAG eyes. However, the rates of VF and cpRNFLT progression were comparable between PICC eyes and their contralateral non-PICC eyes, suggesting that patients who develop PICC tend to have relatively modest longitudinal changes (Figure 3).

All PICC lesions in the present study were located in the inferior region, which is consistent with our previous report showing that most PICCs develop in the temporal to inferior sectors [19]. To evaluate the location-specific impact of PICC, we analyzed VF and cpRNFLT progression separately in the superior and inferior hemifields. The PICC-corresponding superior VF and inferior cpRNFLT showed slower progression in PICC eyes than in their contralateral eyes, and the proportion of eyes with significant superior VF progression was approximately half in PICC eyes compared with their contralateral eyes, although the sample size limited our statistical power. Consistent with this, our multivariable models showed that the presence of PICC was associated with slower progression in the corresponding superior VF. We recently reported that myopia-related tissue disruption and cpRNFLT thinning contribute to localized VF sensitivity reduction in the PICC region [19]. These characteristics imply that PICC-related VF impairment may share features of myopic optic neuropathy (MON), in which defects often arise from structural deformation rather than IOP-related axonal loss. Some previous studies suggest that VFDs linked to myopic structural abnormalities may demonstrate slow or little progression [22,42]. In line with these findings, the modest deterioration observed in the PICC region in our cohort supports the possibility that VFDs associated with PICC follow a clinical course that differs from that of typical glaucoma.

The mechanisms underlying the association between PICC and slower corresponding VF progression may have several explanations. One possibility is that PICC-associated VFDs may occur in an event-like manner, similar to the pattern reported for VFDs associated with optic disk tilt [22], where a localized VFD develops once a certain degree of structural deformation is reached and then remains relatively stable over time. Another possibility is that disruption of the border tissue and peripapillary scleral deformation in PICC eyes [43] alters the biomechanical environment of the ONH. Structural discontinuities may redistribute mechanical or IOP-related stresses on the lamina cribrosa, which could contribute to slower VF progression. These hypotheses remain speculative, and further comparison with typical glaucomatous cases without PICC is needed to clarify how PICC-associated changes interact with glaucomatous mechanisms.

PICC eyes demonstrated marked myopic ONH structural changes consistent with our previous report [20], and multivariable models showed that greater SFO/BMO offset magnitude, reflecting more extensive enlargement of the PPA-gamma and delta zones, was associated with faster superior VF progression. Previous studies have also indicated that enlargement of the PPA-gamma beyond a certain threshold becomes increasingly related to functional loss in HM eyes [44]. The inferior temporal peripapillary region is known to be especially susceptible to myopia-related structural deformation [11,43], which may explain why enlargement of the PPA-gamma and delta zones in this region contributes to the progression of the superior VF. These observations suggest that focal cavitation itself and extensive peripapillary deformation may have distinct and sometimes opposing influences on the clinical course of VFDs in PICC eyes, highlighting the complex interplay of structural factors in myopic OAG.

**Figure 4:**
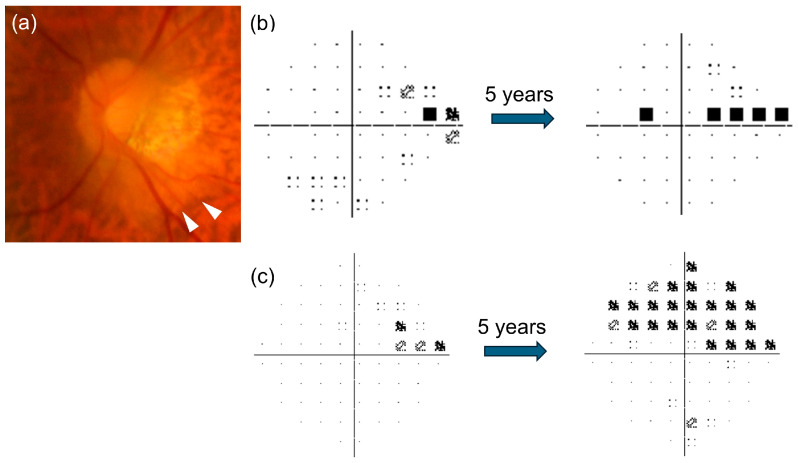
(**a**) Fundus photograph of an eye with peripapillary intrachoroidal cavitation (PICC, white arrowheads) and the pattern deviation plots of the (**b**) 24-2 and (**c**) 10-2 Humphrey visual field tests at baseline (**left**) and after 5-year follow-up (**right**). Significant visual field progression was observed in the superior hemifield corresponding to the inferior PICC.

Although more than 90% of PICC eyes did not exhibit significant progression in the corresponding superior VF, two eyes in our cohort demonstrated significant progression (Figure 4). These eyes also showed significant progression on 10–2 VF, as well as exhibited smaller BMO areas (2.41 mm^2^, 2.62 mm^2^) and relatively smaller SFO areas (2.87 mm^2^, 2.60 mm^2^) compared with other PICC eyes. These ONH configurations may be associated with a reduced neural canal minimum cross-sectional area (NCMCA), which reflects the minimum space available for axons to pass through the neural canal [30]. A recent study demonstrated that a smaller NCMCA was significantly associated with faster VF progression and that HM-associated nasal tilting accelerated temporal glaucomatous damage [21]. In addition, both eyes were at a relatively early glaucoma stage (baseline MD: −1.25 dB, −2.65 dB), raising the possibility that VF deterioration may have been more detectable during the follow-up period. These findings suggest that PICC eyes with a smaller neural canal or at earlier glaucoma stages may be susceptible to VF worsening, warranting careful long-term monitoring, including central VF assessment.

There are some limitations to our study. First, the analysis was restricted to OAG patients with unilateral PICC. In our prior study of consecutive PICC cases, bilateral PICC was identified in 36 of 93 patients (38%) [19]. Although limiting the cohort to unilateral cases may introduce selection bias, this design reduces interparticipant variability and allows more precise characterization of structural features attributable to PICC. Second, the limited sample size may have reduced the statistical power to detect differences between PICC eyes and contralateral eyes. In addition, given the modest progression observed in our cohort, a follow-up period of approximately 5 years may be insufficient to capture clinically meaningful progression. Further studies with larger cohorts and extended follow-up durations are needed to validate our findings and to clarify the longitudinal characteristics of OAG eyes with PICC. Another limitation is that it was not possible to fully separate the independent effects of PICC from glaucomatous damage. Future longitudinal studies including PICC eyes without glaucoma will be important to further understand the natural history and clinical impact of PICC itself. Furthermore, although retinal and other ocular diseases were carefully excluded, the potential influence of systemic chronic diseases was not specifically evaluated and may represent a source of residual confounding.

## 5. Conclusions

OAG eyes with PICC showed modest structural and functional progression over 5 years, comparable to that in their contralateral non-PICC eyes. The presence of PICC was independently associated with slower progression in the corresponding superior VF, whereas greater myopia-associated structural change was related to faster progression. Although progression was generally modest in PICC eyes, clinically relevant deterioration was observed in a subset of eyes, underscoring the need for careful longitudinal follow-up and detailed evaluation of ONH structure in myopic OAG.

## Figures and Tables

**Table 1:** Baseline characteristics in participants with unilateral peripapillary intrachoroidal cavitation.

	PICC Eyes (*n* = 30)	Contralateral Eyes (*n* = 30)	*p* Value
Age (years old)	54.0 ± 10.2		data
Sex (male/female)	14/16		
IOP (mmHg)	13.53 ± 1.72	13.73 ± 1.95	0.340 *
AL (mm) (range)	26.84 ± 1.22 (24.96, 31.28)	26.56 ± 1.32 (23.1, 30.13)	**0.010** *
Visual acuity (log MAR)	−0.05 ± 0.05	−0.06 ± 0.04	0.935 *
Optic nerve head parameters			
BMO area (mm^2^)	3.51 ± 0.95	2.93 ± 0.87	**<0.001** *
SFO area (mm^2^)	3.01 ± 0.51	2.85 ± 0.61	0.075 ^†^
SFO/BMO offset magnitude (μm)	641.57 ± 189.34	453.34 ± 185.40	**<0.001** ^†^
Disk tilt	1.42 ± 0.18	1.29 ± 0.16	**<0.001** ^†^
Disk rotation (degree)	23.85 ± 17.53	17.16 ± 13.34	0.082 ^†^
Visual field			
Mean deviation (dB)	−4.55 ± 4.19	−4.36 ± 5.65	0.887 *
Superior TD (dB)	−4.81 ± 5.10	−5.70 ± 7.83	0.730 *
Inferior TD (dB)	−4.43 ± 4.91	−3.52 ± 5.34	0.477 *
Structural parameters			
Global cpRNFLT (μm)	84.61 ± 14.11	85.75 ± 15.12	0.590 ^†^
Superior cpRNFLT (µm)	88.88 ± 14.31	90.74 ± 15.46	0.468 ^†^
Inferior cpRNFLT (µm)	80.34 ± 15.84	80.77 ± 17.46	0.777 *
Global cpChT (μm)	109.15 ± 33.14	107.97 ± 34.22	0.685 *
PICC location (superior/inferior)	0/30		

* Wilcoxon signed-rank test. ^†^ Paired *t*-test. Statistically significant results are indicated in bold. PICC: peripapillary intrachoroidal cavitation; IOP: intraocular pressure; AL: axial length; log MAR: log minimum angle of resolution; BMO: Bruch’s membrane opening; SFO: scleral flange opening; TD: total deviation; cpRNFLT: circumpapillary retinal nerve fiber layer thickness; and cpChT: circumpapillary choroidal thickness.

**Table 2:** Comparison of functional and structural progression in participants with unilateral peripapillary intrachoroidal cavitation.

	PICC Eyes (*n* = 30)	Contralateral Eyes (*n* = 30)	*p* Value
Visual field progression (dB/y)			
MD slope	−0.24 ± 0.35	−0.35 ± 0.53	0.271 *
Superior TD slope	−0.22 ± 0.41	−0.38 ± 0.70	0.253 *
Inferior TD slope	−0.29 ± 0.41	−0.32 ± 0.51	0.641 *
Number of eyes with progression			
MD	4 (13.3%)	3 (10.0%)	1.000 ^†^
Superior TD	2 (6.7%)	4 (13.3%)	0.617 ^†^
Inferior TD	2 (6.7%)	3 (10.0%)	1.000 ^†^
Rate of cpRNFLT thinning (µm/y)			
Global	−0.15 ± 1.17	−0.11 ± 0.91	0.909 ^‡^
Superior	−0.54 ± 1.41	−0.04 ± 1.06	0.201 ^‡^
Inferior	0.24 ± 1.40	−0.18 ± 1.43	0.386 *

* Wilcoxon signed-rank test. ^†^ McNemar test. ^‡^ Paired *t*-test. Statistically significant results are indicated in bold. PICC: peripapillary intrachoroidal cavitation; MD: mean deviation; TD: total deviation; and cpRNFLT: circumpapillary retinal nerve fiber layer thickness.

**Table 3:** Univariable and multivariable associations with superior and inferior total deviation slope.

	Superior TD Slope				Inferior TD Slope			
	Univariable		Multivariable		Univariable		Multivariable	
	Standardized Estimate	*p* Value *	Standardized Estimate	*p* Value *	Standardized Estimate	*p* Value *	Standardized Estimate	*p* Value *
Age (years old)	0.17 ± 0.13	0.203			0.20 ± 0.15	0.183	0.23 ± 0.13	0.099
Sex (female = 1)	−0.09 ± 0.13	0.485			−0.24 ± 0.15	0.103	−0.28 ± 0.13	**0.042**
IOP (mmHg)	0.06 ± 0.13	0.645			−0.14 ± 0.14	0.321		
AL (mm)	0.07 ± 0.13	0.589			0.19 ± 0.14	0.184		
BMO area (mm^2^)	−0.07 ± 0.13	0.573			−0.06 ± 0.13	0.652		
SFO area (mm^2^)	−0.04 ± 0.13	0.768			−0.13 ± 0.13	0.330		
SFO/BMO offset magnitude (μm)	−0.14 ± 0.13	0.286	−0.28 ± 0.14	**0.047**	−0.02 ± 0.12	0.860		
Disk tilt	−0.02 ± 0.13	0.869			0.07 ± 0.13	0.568		
Disk rotation (degree)	−0.13 ± 0.13	0.312			0.00 ± 0.12	0.982		
Baseline TD (dB)	−0.16 ± 0.13	0.208	−0.21 ± 0.12	0.099	0.17 ± 0.12	0.191		
Baseline cpRNFLT (µm)	−0.02 ± 0.13	0.858			0.43 ± 0.12	**0.001**	0.45 ± 0.12	**<0.001**
Presence of PICC	0.14 ± 0.12	0.260	0.29 ± 0.13	**0.037**				

* Linear mixed models. Statistically significant results are indicated in bold. PICC: peripapillary intrachoroidal cavitation; IOP: intraocular pressure; AL: axial length; BMO: Bruch’s membrane opening; SFO: scleral flange opening; TD: total deviation; and cpRNFLT: circumpapillary retinal nerve fiber layer thickness.

## Data Availability

The raw data supporting the conclusions of this article will be made available by the authors on request.

## Abbreviations

The following abbreviations are used in this manuscript:

OAG	open-angle glaucoma
HM	high myopia
VF	visual field
ONH	optic nerve head
AL	axial length
IOP	intraocular pressure
GON	glaucomatous optic neuropathy
PICC	peripapillary intrachoroidal cavitation
OCT	optical coherence tomography
VFDs	visual field defects
cpRNFLT	circumpapillary retinal nerve fiber layer thickness
BMO	Bruch’s membrane opening
PPA	peripapillary atrophy
SFO	scleral flange opening
ASCO	anterior scleral canal opening
BT	border tissue
cpChT	circumpapillary choroidal thickness
MD	mean deviation
TD	total deviation
NCMCA	neural canal minimum cross-sectional area
